# Planned oocyte cryopreservation in Hong Kong: a potential prototype for mainland China

**DOI:** 10.1080/26410397.2025.2485547

**Published:** 2025-05-25

**Authors:** Olivia MY Ngan, Ernest HY Ng, Yumeng Yue, Raymond HW Li

**Affiliations:** a Research Assistant Professor, Medical Ethics and Humanities Unit, School of Clinical Medicine, LKS Faculty of Medicine, The University of Hong Kong, Hong Kong; Research Fellow, Centre for Medical Ethics and Law, Faculty of Law and LKS Faculty of Medicine, The University of Hong Kong, Hong Kong. *Correspondence*: olivian1@hku.hk; b Clinical Professor, Department of Obstetrics and Gynaecology, School of Clinical Medicine, Li Ka Shing Faculty of Medicine, The University of Hong Kong, Hong Kong; c Research Assistant, The Bau Institute of Medical and Health Sciences Education, Li Ka Shing Faculty of Medicine, The University of Hong Kong, Hong Kong; d Clinical Associate Professor, Department of Obstetrics and Gynaecology, School of Clinical Medicine, Li Ka Shing Faculty of Medicine, The University of Hong Kong, Hong Kong

**Keywords:** planned oocyte cryopreservation, fertility preservation, Hong Kong, China

## Abstract

The advent of planned oocyte cryopreservation (planned OC) represents a pivotal transition from reactive infertility treatments to proactive fertility preservation, providing a contemporary solution for women aiming to synchronise their career aspirations with future fertility plans. While numerous developed Western nations have liberalised access to planned OC for diverse individuals, including opposite-sex married couples, same-sex married couples, and unmarried individuals, mainland China maintains stringent prohibitions, permitting it solely for medical reasons, due to medical, cultural, and ethical considerations. In contrast, Hong Kong, a major urban city in China, has adopted a more permissive approach, allowing access to planned OC for non-medical reasons among unmarried individuals. This article will delve into the evolving landscape of public attitudes, fertility-seeking behaviour, and regulatory governance in Hong Kong. It will reflect on the practices and challenges associated with implementing a more permissive policy on planned OC, aiming to extract valuable lessons for the broader Chinese context.

## Plain language summary

Reproductive rights are a key part of human rights. They enable people to make their own choices about their reproductive health and family planning. Fertility preservation, like freezing eggs, is an integral part of these rights, helping individuals have children when ready. While many Western countries offer accessible clinical services for planned oocyte cryopreservation, mainland China has strict rules, especially for individuals, due to medical, cultural, and ethical reasons. Hong Kong, an urban city that shares cultural values with mainland China, operates under different legal jurisdictions concerning reproductive technologies. This review examines how Hong Kong’s regulatory governance of planned oocyte cryopreservation and public attitudes support a rights-based approach. It demonstrates how a rights-based framework can effectively support national birth rate objectives while seamlessly integrating technological advancements with cultural and ethical considerations.

## Résumé

L’avènement de la cryoconservation planifiée des ovocytes représente une transition cruciale de traitements réactifs de l’infertilité en faveur de la préservation active de la fertilité, offrant une solution contemporaine pour les femmes qui souhaitent synchroniser leurs aspirations professionnelles avec leurs projets futurs de maternité. Si de nombreux pays occidentaux développés ont libéralisé l’accès à la cryopréservation planifiée d’ovocytes pour divers groupes de personnes, notamment les couples mariés de sexe opposé, les couples mariés de même sexe et les célibataires, la Chine continentale maintient de strictes interdictions, l’autorisant uniquement pour des raisons médicales, du fait de considérations médicales, culturelles et éthiques. En revanche, Hong Kong, grande ville urbaine de Chine, a adopté une approche plus permissive, autorisant l’accès à cette procédure pour des raisons non médicales aux personnes célibataires. Cet article se penchera sur l’évolution des attitudes du public, du comportement de recherche de la fécondité et de la gouvernance réglementaire à Hong Kong. Il réfléchira aux pratiques et aux défis associés à la mise en œuvre d’une politique plus permissive de cryoconservation planifiée des ovocytes, dans le but d’en tirer des enseignements précieux pour le contexte chinois plus large.

## Resumen

El advenimiento de la criopreservación planificada de ovocitos (CPO) representa una transición fundamental desde los tratamientos de infertilidad reactivos hacia la preservación de fertilidad proactiva, lo que ofrece una solución contemporánea para las mujeres que buscan sincronizar sus aspiraciones profesionales con futuros planes de fertilidad. Si bien numerosas naciones occidentales desarrolladas tienen acceso liberalizado a la CPO para diversas personas, incluidas las parejas casadas de sexos opuestos, parejas casadas del mismo sexo y personas solteras, China continental mantiene prohibiciones estrictas que permiten la CPO exclusivamente por razones médicas, debido a consideraciones médicas, culturales y éticas. En cambio, Hong Kong, una ciudad urbana principal de China, ha adoptado un enfoque más permisivo, que permite el acceso a la CPO por razones no médicas entre personas solteras. Este artículo ahondará en el panorama en evolución de actitudes públicas, comportamientos de búsqueda de fertilidad y gobernanza regulatoria en Hong Kong. Reflexionará sobre las prácticas y los retos asociados con aplicar una política más permisiva relativa a la CPO, con el objetivo de extraer lecciones valiosas para el contexto chino en general.

## Background

Oocyte cryopreservation (OC), introduced as an experimental medical intervention in the late 1980s, was primarily utilised to preserve fertility in women undergoing medical treatments that could impair their reproductive abilities.^1^ The success rates of OC have improved significantly with advances in vitrification techniques, making it a viable option for women juggling motherhood and modern life, reshaping reproductive timing even without medical indications.^2^ Such a procedure performed for non-medical or social reasons is called planned oocyte cryopreservation (planned OC).^3^

Epidemiological studies demonstrate that a woman's natural fertility gradually declines after age 30, with a sharper drop after age 35,^4^ reducing the likelihood of conception^5,6^ and coinciding with an increased risk of chromosomal abnormalities^7^ in offspring due to the diminishing quantity and quality of oocytes. Planned OC emerges as a fertility reassurance against these age-related challenges for future childbearing.^8^ A narrative review by Walker et al. provided compelling evidence for the importance of egg quality in successful outcomes, demonstrating a significantly higher chance of future pregnancy for women who froze their eggs at a younger age than those of advanced maternal age.^9^ The demands of modern society, particularly the pursuit of personal and professional aspirations, often compel women of childbearing age to postpone motherhood but without necessarily sacrificing reproductive potential.^10–13^^.^

### Current situation in mainland China and Hong Kong

Within Western feminist and policy discourse, which is grounded in frameworks emphasising individual autonomy, planned OC is endorsed as a means of empowering reproductive choice.^14^ Conversely, Eastern societies perceive planned OC as a public health and social strategy aimed at mitigating persistently low birth rates and safeguarding societal sustainability and public interests.^15,16^

At present, mainland China permits OC for medical reasons but prohibits it for non-medical purposes.^17^ A landmark case set a significant precedent when a single woman sued the hospital after being denied access to planned OC, igniting ongoing discussions about reproductive rights and gender discrimination in China.^18^ The National Health Commission commands that assisted reproductive institutions and practitioners must abide by national laws and regulations and relevant codes of conduct.^18^ However, there is a prevailing expectation that Chinese regulatory bodies may consider relaxing restrictions on planned OC practices in the next five years, signalling a forthcoming paradigm shift in policy orientation to better align with the evolving demographic landscape.^19^

Hong Kong, as part of China, though running on separate policies, adopts a permissive stance and emphasises procedural safety,^20^ with single women allowed access to planned OC for non-medical reasons but only through the private sector (no public subsidies are available). This approach does not differentiate between medical and non-medical reasons, emphasising individual choice and reflecting broader neoliberal principles prioritising personal responsibility and market-driven solutions. Hong Kong could serve as an initial model to build upon, offering valuable lessons and providing a foundational framework for addressing similar demographic issues in mainland China.

This article aims to elicit the evolving landscape of public attitudes, fertility-seeking behaviour, and the governing framework in Hong Kong. By examining the practices and challenges faced by Hong Kong in implementing a more permissive policy on planned OC, this article seeks to extract valuable lessons that can be applied to the broader Chinese context. Understanding how Hong Kong negotiates the balance between modern reproductive technologies and traditional cultural values can provide a potential prototype for addressing similar issues within mainland China.

### Positionality statement

The research team consists of two Chinese females (OMYN and YY) and two Chinese males (EHYN and RHWL). OMYN is a researcher with a public health and ethics background, with a primary interest in reproductive technologies ethics. EHYN and RHWL are clinicians and researchers, each working in a tertiary assisted reproduction unit in Hong Kong, providing the full range of assisted reproduction including planned OC and conducting laboratory/clinical research. YY is a research assistant with a background in public health and nursing, and her research interests include health policy and reproductive health. When the manuscript for this article was drafted, planned OC was not yet legalised in mainland China.

## From reactive infertility treatment to seeking proactive fertility preservation: evolving childlessness culture

### The enduring influence of Confucian values: filial piety and procreation

The pressure to procreate remains deeply embedded within Chinese society, heavily influenced by Confucian ideals and the concept of filial piety.^21,22^ Traditionally, Chinese society emphasises the importance of family and the value of having children, particularly sons, who are crucial for perpetuating the family lineage. Bearing children is viewed as a fundamental familial obligation, and childlessness can carry a significant cultural stigma.^23^ Historically, an inability to produce offspring, especially sons, brought immense social shame to couples in China, with the burden often placed on the wife. Traditional practices even permitted husbands to take additional wives or concubines solely to secure descendants, highlighting the immense pressure to fulfil this cultural imperative.^24^ Some Western scholars have viewed this traditional Chinese marital structure as a form of polygamy.^25^ It was not until 1950 that monogamy became mainland China's sole legal form of marriage,^26^ with Hong Kong following suit in 1971.^27^

The cultural significance of fertility and motherhood in Chinese communities is closely intertwined with gender identity. The burden of infertility traditionally falls more heavily on women, reflecting deeply ingrained cultural norms regarding gender roles and reproduction within the patrilineal inheritance system.^28^ Women are more likely to internalise blame for failing to conceive, leading to feelings of guilt, inadequacy, and significant emotional distress.^29^ The societal expectation for women to bear children creates a situation where the wife in an infertile couple can be subjected to intense pressure and scrutiny, particularly from the husband's family.^29,30^ This scrutiny often leads to strained relationships with in-laws.^31^ Women may become the target of blame and criticism, which can even lead to marital discord and, tragically, suicide among young married women. Infertile women encounter more stress and a lower quality of life than their husbands.^32^ A recent study showed that parental concerns, such as father-absent parenting and an unequal gender family and social culture, inhibit fertility intention among the Chinese population.^33^

### Involuntary childlessness and treatment-seeking patterns

Despite the relatively high prevalence of infertility in Hong Kong, with estimates suggesting one in six to seven couples of childbearing age experiencing difficulties conceiving, there appears to be a lack of public awareness and a predominantly reactive approach to seeking treatment. A cross-sectional study by Leong (2001), involving over 7000 respondents, identified a significant gap between the percentage of individuals experiencing fertility issues and those actively undergoing or having undergone treatment.^34^ Among those, 1,153 (16%) reported fertility issues, yet only 34% of those affected had received or were undergoing fertility treatment. This indicates that a significant portion of individuals affected by infertility do not actively seek or pursue medical interventions. Further evidence supporting this trend comes from a more recent study.^35^ Among women actively trying to conceive, a concerning number (40%) had been attempting for more than a year, exceeding the recommended timeframe for seeking medical evaluation. Additionally, over one-third of these women suspected potential fertility concerns, yet only a handful of the minority had taken proactive steps to seek professional assistance. This prevalent “wait and see” attitude towards infertility contributes to delayed treatment initiation.^36^ Many women rely on natural methods of conception tracking, ovulation monitoring, and timed intercourse, hoping for spontaneous pregnancy without medical intervention. These reactive approaches often result in delays in receiving appropriate medical care. Factors contributing to this phenomenon might include unsupportive partners, embarrassment, fear of disappointment, or unawareness of fertility treatment.^37^

### Shifting tides towards proactive fertility management

Hong Kong's work-centric culture presents a significant challenge for individuals seeking to balance career aspirations with family life.^38^ Increased educational attainment and employment opportunities for women have demonstrably shifted priorities, leading more women to prioritise career advancement. This trend manifests in a preference for smaller families or even remaining child-free, reflecting a broader cultural shift in how the younger generation prioritises personal independence and self-actualisation over conforming to these family expectations.^39^ Chen et al. also highlighted that the quality of the family environment, which is strongly linked to subjective quality of life, can still impact fertility intentions.^39^

Despite delaying parenthood, this demographic still desires to have children, which is perceived to be important for marriage.^40^ The rising number of individuals, particularly women over 30, choosing to postpone parenthood raises concerns about subfertility and the potential difficulties associated with conceiving later in life. With greater access to information and evolving social norms, couples adopt a more proactive approach to fertility preservation and management. Recent data from the Hong Kong Council on Human Reproductive Technology indicate a significant increase in access to assisted reproductive technologies (ART), specifically IVF treatment cycles, performed on women aged 35 and above,^41^ which signifies a growing recognition of the importance of proactive fertility management. This shift from reactive to proactive approaches in fertility management represents a significant paradigm change in family planning perceptions. Individuals are becoming more aware of the challenges associated with delayed parenthood and are taking a more active role in safeguarding their reproductive health.^42^

A crucial challenge in proactive fertility management is public ignorance regarding the impact of age on fertility and the success rates of subfertility treatments. While a significant portion of the population acknowledges the decline in treatment success rates with advancing female age, a concerning number believe that ART can entirely negate the effects of ageing.^43^ Another study has also indicated a deficiency in knowledge and awareness of fertility preservation among healthcare practitioners from various disciplines, such as oncology, paediatrics, and surgery.^44^ These knowledge gaps highlight the urgent need for comprehensive educational and awareness campaigns to bridge the gap in understanding subfertility and the limitations of reproductive technologies. Equipping individuals with accurate information about the impact of age on fertility and realistic expectations of ART is crucial for informed decision-making.

## Hong Kong regulatory governance: practices and reflections

The regulation of gamete and embryo donation is meticulously overseen by the Hong Kong Council on Human Reproductive Technology, ensuring compliance with stringent ethical standards and legal requirements.^20^ The Code of Practice offers detailed guidance on institution licensing, quality and safety, informed consent, data protection, and ethics compliance, ensuring all licensed clinics and practitioners adhere to strict ethical, safety, and quality standards.^45^ Topics covered in the Code of Conduct include regulatory operations, and we describe below our major practices and reflections:

### How long can unfertilised eggs be stored?

Hong Kong’s current policy dictates a maximum storage period of 10 years or until the woman reaches 55 years old (whichever comes later) for medically necessary procedures. However, the storage period is strictly capped at 10 years for non-medical reasons.

#### Current debate on the stringent storage period

There are arguments suggesting that the short storage period for planned OC has become outdated and fails to meet modern society’s diverse needs and lifestyles, particularly amidst rapid developments in reproductive technology. For instance, if a woman freezes her eggs in her mid-20s, the institution must discard them once she reaches her mid-30s and she must undergo the procedure again. This requirement seems unreasonable, as discarding viable eggs forces women to undergo repeated intrusive and uncomfortable medical procedures, such as drug-induced ovarian stimulation and oocyte retrieval, which carry certain risks. Moreover, the quality of women’s eggs diminishes with advancing age, rendering them inferior to those produced during her 20s. Some critics advise the government to increase the duration of storage for planned OC, arguing that such an extension would further support women in aligning their reproductive planning with their broader life trajectories and balancing other medical risks.^46^ From a clinical perspective, the policy on oocyte storage duration, whether limited to 10 years or extended indefinitely, often shows little difference in clinical outcomes. However, procedural fairness in storage and discarding procedures is a compelling aspect that necessitates policy discussion. The American Society for Reproductive Medicine (ASRM) highlights that planned OC is ethically permissible and serves legitimate interests in reproductive autonomy while recognising its long-term efficacy and safety uncertainties.^8^ Ethical and legal challenges often arise from the lack of clear regulations and the potential for disputes over the ownership and disposition of stored oocytes. Policies must also address the implications of long-term storage, such as fee dispute and discard procedures when payment is not settled or couples are separated.

### Who and when can have access to the stored gametes?

Single women are permitted to undergo planned OC at approved clinics. However, the utilisation of the stored gametes is contingent upon the woman being married, as Hong Kong's legal framework restricts access to the use of stored gametes to parties within legally recognised opposite-sex civil unions. This policy contrasts sharply with practices in some Western countries, such as the United States, where there has been a notable increase in single women conceiving through sperm banks or informal sperm donation to achieve pregnancy.^47^ Additionally, it is stipulated that upon a woman's death, her stored oocytes should not be used by her spouse to bring about any posthumous children.

#### Concerns about compromising Chinese values through non-marital childrearing

Beyond a legal contract, marriage symbolises a mutual dedication to nurturing and supporting offspring together. The regulation restricting unmarried individuals’ access to gametes in Hong Kong is designed to prevent non-marital childbearing through ART, thus adhering to traditional Chinese values. This policy is not necessarily an act of discrimination but rather an alignment with cultural norms that have shaped Hong Kong’s societal values and traditions. It reflects the influence of Confucian ideals on parenthood, emphasising the significance of family and marriage as the cornerstone of society.^30^ By permitting planned OC, this practice harmonises with cultural values by allowing women to postpone childbearing without compromising their ability to have biological children in the future, thereby potentially strengthening lineage. Moreover, restricting access to reproductive technologies for unmarried individuals helps to prevent non-marital childbearing, thereby promoting societal stability and preserving the traditional family structure that is integral to the cultural fabric. This aligns with a cultural emphasis on the family unit.

The ban on posthumous reproductive technologies also underscores the belief that marriage provides a stable and nurturing environment essential for child development and well-being. Prohibiting posthumous reproduction is crucial in maintaining the traditional essence of marriage and family dynamics. When one partner passes away, the dynamic of this partnership is irrevocably changed. Considering the paramount importance of the child’s welfare, Hong Kong is against permitting the posthumous use of gametes or embryos by the surviving spouse. Hong Kong’s approach is culturally sensitive and resonates with the values prevalent in mainland China, integrating traditional family planning norms into the contemporary context of marriage and lifestyle adaptations.

### Who can donate unused materials, and in what forms?

Other than self-use, individuals may consent to donate altruistically their gametes or embryos to other infertile couples, aiding those struggling with infertility to achieve pregnancy. Egg donors must be between the ages of 18 and 34, in good health, and free from any personal or family history of genetic disorders or records of abnormal births. It is preferred that egg donors have had their own biological children before the egg donation, it is not a mandatory requirement. To mitigate the potential risk of incest, guidelines stipulate that gametes or embryos from any single donor should not be used to produce more than three live birth events or fewer, depending on the donor’s consent. This limitation is crucial in preventing many offspring from a single donor.

#### Practical challenges in the absence of local-wide egg banks

Local interest in oocyte or embryo donation as a means to address infertility is minimal.^40^ Hong Kong does not have egg banks to assist in matching egg donors and recipients. The most common method is to ask relatives or friends to see if anyone is willing to be a designated donor. Asking relatives or friends to donate eggs can be particularly challenging due to the personal and sensitive nature of the request.^48^ This can create emotional complexity, as requesting such a significant favour from someone close can strain personal relationships. Privacy concerns also arise, as both the donor and recipient might worry about the implications of the donation on their relationship. Additionally, cultural sensitivity plays a role, as discussing fertility and reproductive issues can be particularly private matters in many cultures, including those influenced by traditional Chinese values. Ethical considerations further complicate the situation, as potential donors may feel pressured or obligated, especially if they are close relatives or friends. These factors highlight the challenges and sensitivities involved in the current system in Hong Kong, where egg donation relies heavily on personal connections rather than anonymous donations through egg banks.

### What is the age limitation for accessing ART?

Hong Kong currently does not impose explicit age restrictions on access to ART. However, attempting IVF with one's eggs is not recommended for women over the age of 45, as the chances of conceiving and having a baby using their eggs at that age are very low. Local data indicates little ART access for women over 50, suggesting a low interest in using ART among the advanced maternal-age population.^41^

#### Health concerns of post-menopausal mothers

International media have highlighted a growing trend of post-menopausal women achieving pregnancy through previously frozen or donor eggs with the aid of ART.^49^ In response to clinical and ethical concerns, some governments have implemented legal bans on IVF for women above a certain age and have also set age restrictions for egg freezing, acknowledging the decline in egg quality with age,^50,51^ balancing between reproductive autonomy and the potential health risks involved in advanced maternal-age pregnancies.

Based on our experience with the Chinese population, requests for oocyte donation from women of advanced maternal age are relatively uncommon in Hong Kong despite the absence of an upper age limit for ART in the region. Obstetricians will advise against embryo replacement after menopause due to the significantly elevated obstetric risks. According to the ASRM,^52^ while oocyte donation can mitigate age-related declines in implantation and birth rates, it does not eliminate the heightened risk of obstetrical complications in older patients. These complications include increased rates of operative delivery, hypertensive disorders, gestational diabetes, and perinatal mortality. Doctors are encouraged to conduct thorough medical evaluations to assess the physical fitness of patients for pregnancy before proceeding with embryo transfer in women of advanced reproductive age. The ASRM guidelines strongly discourage donor oocytes in women over the age of 55 or those with underlying medical conditions that exacerbate obstetrical risks.^52^ This cautious approach is essential to ensure the safety and well-being of both the mother and the potential offspring.

### Why is planned OC not offered under a governmental-subsidised healthcare system?

Hong Kong adopts a two-tiered healthcare system characterised by the public sector that supports affordable health services and the private sector offering various healthcare options at self-expense. Elective procedures like planned OC are provided outside the government-subsidised healthcare system due to two factors – medical necessity and resource allocation. Public healthcare systems prioritise medical necessity and resource allocation when determining coverage. Fertility preservation for medical reasons, such as before cancer treatment, is typically covered because it protects future fertility. In contrast, elective procedures like planned OC may not meet the criteria for medical necessity, ensuring resources address the most pressing health needs. Resource allocation focuses on treatments and services that address immediate and critical health needs. Given finite resources, public healthcare systems prioritise essential medical services with a direct impact on public health. Planned OC, often considered elective for non-medical reasons, may not be seen as crucial compared to emergency care and preventive health measures. Ethical considerations about fair resource distribution lead policymakers to be cautious about allocating resources to elective, non-medically necessary procedures.

Operating planned OC within a privatised market is clinically and ethically justifiable. This approach allows for the allocation of public healthcare resources to more immediate and critical health needs while still providing individuals with the opportunity to pursue fertility preservation through private means. Clinically, it ensures that elective procedures do not burden the public healthcare system, thereby maintaining its focus on essential medical services. Ethically, it respects individual autonomy and the right to make personal reproductive choices while acknowledging the importance of fair resource distribution within the public sector.

#### Ethical concerns for privatised planned OC at private clinics

The high cost of planned OC in the private sector in Hong Kong can represent a substantial personal burden and raise health inequity due to access barriers. This financial strain is particularly frustrating for those who view OC as their last hope to preserve the desire to have biological children. The emotional and psychological toll of age-related infertility is immense, and the added pressure of navigating financial barriers to access this service can be overwhelming. Many women find themselves in a difficult position, having to weigh their desire for future motherhood against significant costs, often without the support of public healthcare options. This situation underscores the profound challenges and heartache faced by women striving to secure their reproductive futures.

When a country like China bans planned OC, despite significant interest among Chinese women, the public health implications extend beyond individual reproductive choices. One of the most notable consequences is the rise in medical tourism,^53^ as women travel to other cities or countries where the procedure is legal. Medical tourism costs for planned OC, including travel and procedure fees in another city or country, can be considerable. This often restricts access to wealthier individuals, exacerbating social inequalities. Women who opt for medical tourism face logistical and emotional hardships, such as being treated in a foreign healthcare system without the support of friends and family. The quality and safety of procedures may vary, raising concerns about risks and the need for follow-up care. Complications from the OC process can lead to health disadvantages if women receive a different standard of care than they would at home. Communication difficulties, differences in medical standards, and the need for continuous care relationships with healthcare providers are additional obstacles that these women must navigate.

Besides, the trend has far-reaching effects on the individuals involved and the broader public health landscape. From a population health perspective, banning a high-demand service may indirectly affect fertility intentions. If women delay childbearing while waiting for the opportunity to freeze their eggs until such services become available, this could impact maternal age and potentially influence demographic trends.

## Conclusion

Hong Kong’s approach, which does not differentiate between medical and non-medical reasons for OC, exemplifies a model of inclusivity and respect for individual reproductive choices. This policy reflects a progressive alignment with contemporary society’s evolving needs and values, recognising the importance of providing equitable access to fertility preservation for all women, regardless of their reasons for seeking the procedure. We also reflect on the practice and challenges of ensuring that the current regulatory framework remains responsive to societal changes and advancements in reproductive technology. However, it is essential to acknowledge that directly replicating Hong Kong’s policy in mainland China might not be feasible due to the vast differences in population size, socio-economic structures, and cultural contexts. Further policy research is necessary to explore the social and ethical perspectives of various stakeholders in mainland China, including women, medical professionals, and policymakers, and balance preserving cultural values with accommodating contemporary societal needs, ensuring that policies are both culturally sensitive and practically effective.

## Provenance statement

This article was not commissioned and went through external peer review.
